# Molecular Dynamics: Investigating the Self-Association of Stearic Acid and Heteroassociation of Stearic Acid–Water in Cyclohexane

**DOI:** 10.1021/acsomega.3c03473

**Published:** 2023-10-25

**Authors:** Najib Sharifi

**Affiliations:** Institute for Energy and Environmental Flows and Department of Chemistry, University of Cambridge, Cambridge CB2 1EW, U.K.

## Abstract

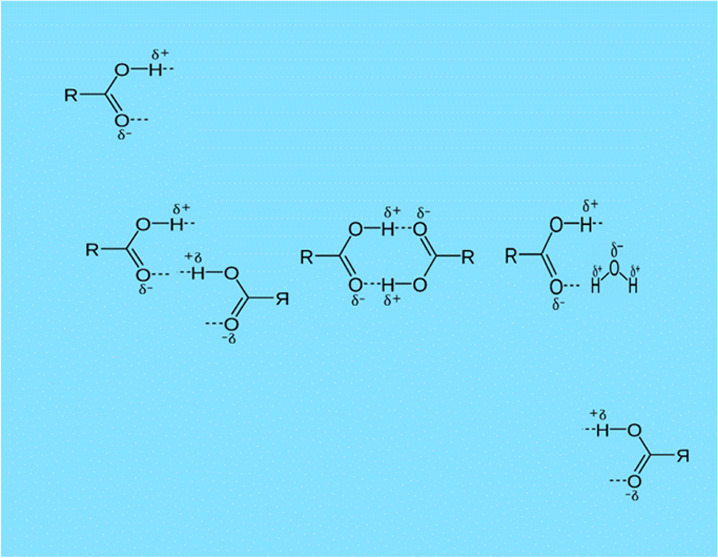

The self-association of molecular additives determines the chemical potential in the bulk and, in turn, the adsorbed amount onto a surface for a number of important commercial applications such as wind turbines. Molecular dynamics simulations have been utilized as a technique to study the self-association of model additive, stearic acid, and heteroassociation of stearic acid–water, in cyclohexane as a function of temperature. Reasonable values of the enthalpies and equilibrium constants were determined for stearic acid in cyclohexane. The role of water, nearly always present in commercial systems, in solution association was also studied to determine the thermodynamics parameters of hydration (i.e., acid–water heteroassociation). There are very few other studies reporting on these important heteroassociation parameters. The association constants and enthalpies of association obtained from molecular dynamics are in good agreement with experimental data in the literature. A combination of Fourier transform infrared (FTIR) data and molecular dynamics simulation results allows the fraction of open dimers (single hydrogen-bonded dimers) to be estimated in cyclohexane (which is not possible from the experimental FTIR data alone). The fraction of open dimers of stearic acid in cyclohexane at room temperature is ∼1.5% at 25 °C and ∼4% at 70 °C.

## 1 Introduction

In many commercial applications such as gear boxes in wind turbines, engine oil in motor vehicles, and joint lubricants in biomedical applications, the additives used are amphiphilic with a polar headgroup and a nonpolar alkyl tail. These additives self-associate, where the nature of these associations depends on the nature of the solvent. Self-association is very important in determining the behavior of the additives in bulk solutions, which, in turn, determines the resultant adsorption onto any surface of interest. This can be considered to reflect the additive chemical potential, which is determined by the monomer concentration. The monomer concentration, i.e., the extent of association, is strongly dependent on the total additive concentration, and at extremely low concentrations, the additives are mostly dissociated, but the extent of self-association increases with concentration as described below. The self-association of additives results in a change in enthalpy and entropy and is therefore temperature-dependent. This has important significance for commercial applications where solid–solid contact results in significant local heating. Characterizing the temperature dependence has been the subject of many experimental approaches but little computational work.

Furthermore, water is essentially always present in commercial systems, and therefore, it is important to consider the role of water in the bulk solution. The water may have several roles such as associating with the polar additives (further reducing the “free” monomer concentration). While the association of acid–water from aqueous solvents has been the subject of many studies reported in the literature,^1−3^ the heterogeneous association of acid–water in a nonaqueous phase has not been reported much in the literature either experimentally (except our recent paper^4^) or computationally. The latter system is important for commercial applications, such as additives in wind turbines.

There are a number of experimental approaches that have been used to quantify solution self-association,^5^ including FTIR,^6−10^ NMR,^11^ dielectric spectroscopy,^12−17^ cryoscopy,^18,19^ ebullioscopy,^20,21^ isopiestic measurements,^22−24^ acid catalysis,^25^ the heat of dilution^26^ and effective molecular weight.^27^ However, the methods that can be used are generally limited by the physical properties of the system under consideration, the concentrations over which the behavior is to be measured, and access to appropriate facilities. Therefore, theoretical and simulation methods are extremely valuable in calculating such thermodynamic parameters. Jaishankar et al.^28^ employed molecular dynamics simulations to study the free energy of self-association of stearic acid in heptane, and hexadecane, at a single temperature. There have been other studies using molecular dynamics to determine the association of proteins in biological systems.^29^ There has been very little work on the temperature dependence of association using molecular dynamics.

**Figure 1:**
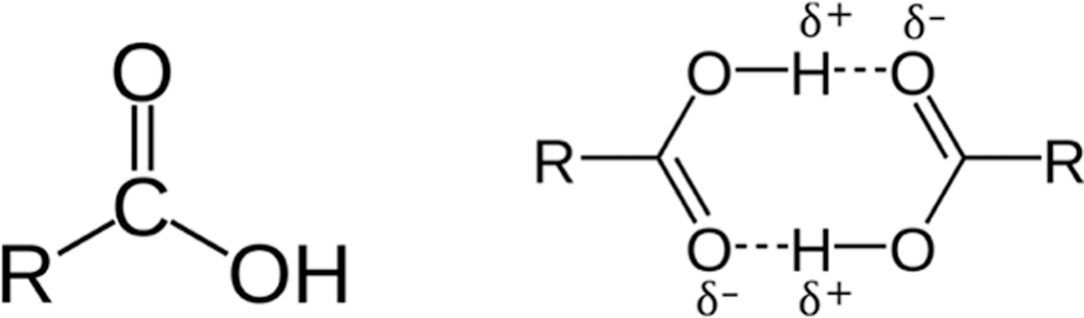
Schematic of a carboxylic acid as a monomer (left) and associated closed dimers formed through hydrogen bonding (right). δ+ and δ− refer to partial charges. Adapted from Farren et al.^4^

## 2 Mathematical Description of Dimerization

A comprehensive mathematical description of solution association can be found in our recent publication,^4^ where a comprehensive experimental and mathematical modeling is presented, or elsewhere in the literature.^30,31^ For carboxylic acids at low concentrations, the monomer and dimer equilibrium in nonpolar solvents can be described by
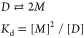
1where *M* is the monomer, *D* is the dimer, and *K*_d_ is the dissociation constant (1/*K*_a_, where *K*_a_ is the association constant). For consistency, we will work with dissociation. However, this equilibrium can be considered as either “association” or “dissociation” with constants interrelated as *K*_d_ = 1/*K*_a_.

This equation can be combined with a mass balance (*C* = *M* + *D*, where *C* is the total acid concentration) to give the monomer concentration^4^ as a function of the total acid concentration, *C*
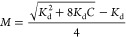
2As previously discussed, water can also associate with the monomer and form the following equilibrium
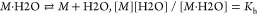
3where *K*_h_ is the heterodissociation constant. Temperature dependence for the dissociation constants was included through the integrated form of the Van’t Hoff equation assuming the enthalpy of dissociation is independent of temperature.
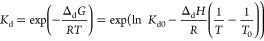
4where *T*_0_ is the reference temperature (25 °C) and *K*_d0_ is the dissociation constant at the reference temperature. Δ_d_*H* and Δ_d_*G* are the enthalpy and Gibbs free energy of dissociation, respectively.

## 3 Simulation Method

The molecular dynamics code, LAMMPS,^32^ was used with two stearic acid molecules (or one stearic acid and one water) dissolved in a cubic box of 1000 cyclohexane molecules. Water molecule bonds were constrained using the SHAKE algorithm.^33^ Periodic boundary conditions were imposed in all 3 dimensions. In all MD calculations, the all-atom L-OPLS-AA force field was used for the organic liquid system^34^ and OPLS-AA for carboxylic functional groups^35^ and SPC^36^ for the water molecule. The L-OPLS-AA force field parameters were obtained from the literature and used without any further refinement.

The system was equilibrated by energy minimization with a subsequent 1 ns NVT simulation with a 0.05 fs time step and a temperature of 1 K, where a Nosé–Hoover thermostat was used to control the temperature. Subsequently, a second NVT simulation was carried out in which the temperature was ramped up to the target temperature (here, simulations were performed at 300, 325, and 345 K) over 2 ns using a 1 fs time step. Furthermore, an NPT simulation was carried out to achieve the correct pressure (1 bar) and hence obtain the correct liquid density, (a bulk density of 814 ± 20 g/L, this value is 6% above the experimental values for cyclohexane and in line with previous simulation work^28,37^).

### 3.1 Calculation of the Potential of Mean Force

**Figure 2:**
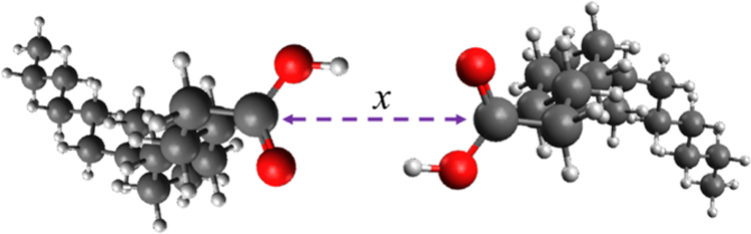
Snapshot of two stearic acid molecules dimerizing as the distance between the two carboxylic head groups is reduced. The distance between the head groups is defined as the distance between the carbonyl carbons. The solvent molecules are not shown for clarity.

The two stearic acid molecules were initially positioned 1.5 nm from each other. The position of the stearic acid molecules is defined by the coordinates of the carbonyl carbon in the fatty acid headgroup (COOH) as shown in Figure 2. The two carbon atoms were slowly pulled together (separation *x*). While the separation distance is defined, the angles and geometries of both molecules are not constrained and are free to change during the simulation. During the pulling stage, a harmonic force constant of 6 kcal/(mol Å^2^) was used to hold the acid groups at a given separation distance. A step size of 0.5 Å was used to draw the molecules together. Configurations, i.e., atomic position snapshots, were stored every 0.1 ps which were subsequently used for umbrella sampling to calculate the free energy using the weighted histogram analysis method (WHAM).^38^ The resultant histograms along the reaction coordinate show good overlap between neighboring histograms and are provided in the Supporting Information.

After each step of 0.5 Å, further equilibration is required, followed by umbrella sampling analysis. A sensitivity analysis was carried out to find the time required for a typical umbrella run that shows good statistical convergence at 2 ns (Supporting Information).

**Figure 3:**
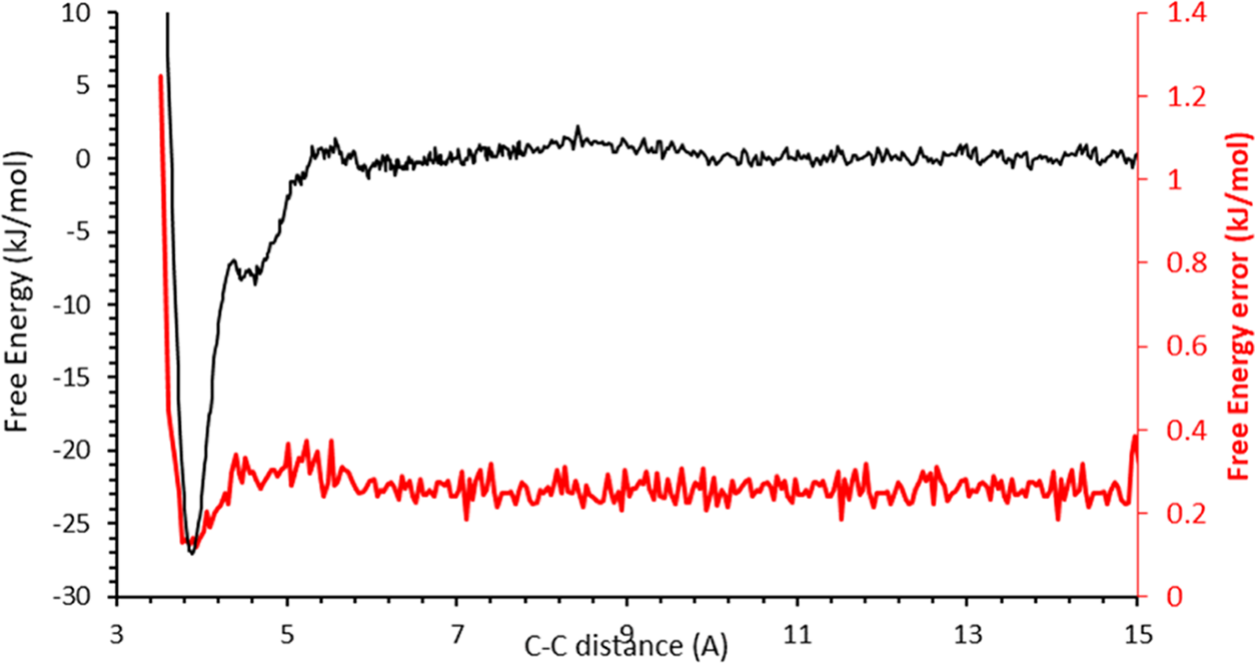
Free energy profile of 2 stearic acid molecules self-associating as they approach each other (300 K). Two minima corresponding to the open and closed dimers are shown. The corresponding error in the free energy as a function of reaction coordinate is also presented.

The free energy profile (and its corresponding error) of two stearic acid molecules approaching each other at 300 K is shown in Figure 3. The free energy profile as a function of the separation distance between the two acid group carbon atoms is essentially constant above 10 Å; therefore, this is taken as the bulk value of the free energy and set to zero. As the two acid groups approach each other, the profile becomes strongly negative with two pronounced minima before sharply rising. The two minima correspond to the open dimer (single hydrogen bond per dimer) and closed dimer (two hydrogen bonds per dimer, Figure 1) cases. The first minimum at 4.6 Å corresponds to a single hydrogen bond between the two acids, i.e., open dimer. The second minimum at 3.8 Å corresponds to two hydrogen bonds between the acid groups, i.e., a closed dimer. The corresponding error in the free energy as a function of the reaction coordinate, estimated from Monte Carlo Bootstrap Error Analysis,^38^ is <1%.

The depths of the free energy minima can be used to approximate the Gibbs free energies of the open and closed dimers. Since there are two states, to get the Gibbs free energy of association, including both the open and closed dimer, one can integrate the over the potential well. The Gibbs free energy is reportedly given by the following relationship:^28^
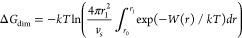
5*r*_0_ = 3.9 Å and *r*_1_ = 5.3 Å define the lower and upper limits of the free energy well (this is obtained from the dimerization range in the free energy profile), respectively. *T* is the temperature, *k* is the Boltzmann constant, and *W*(*r*) is the free energy obtained from WHAM. The equation also includes normalization against the molecular volume of the solvent, *v*_s_, based on the bulk solvent density at the corresponding temperature.

## 4 Results

### 4.1 Stearic Acid in Cyclohexane

**Figure 4:**
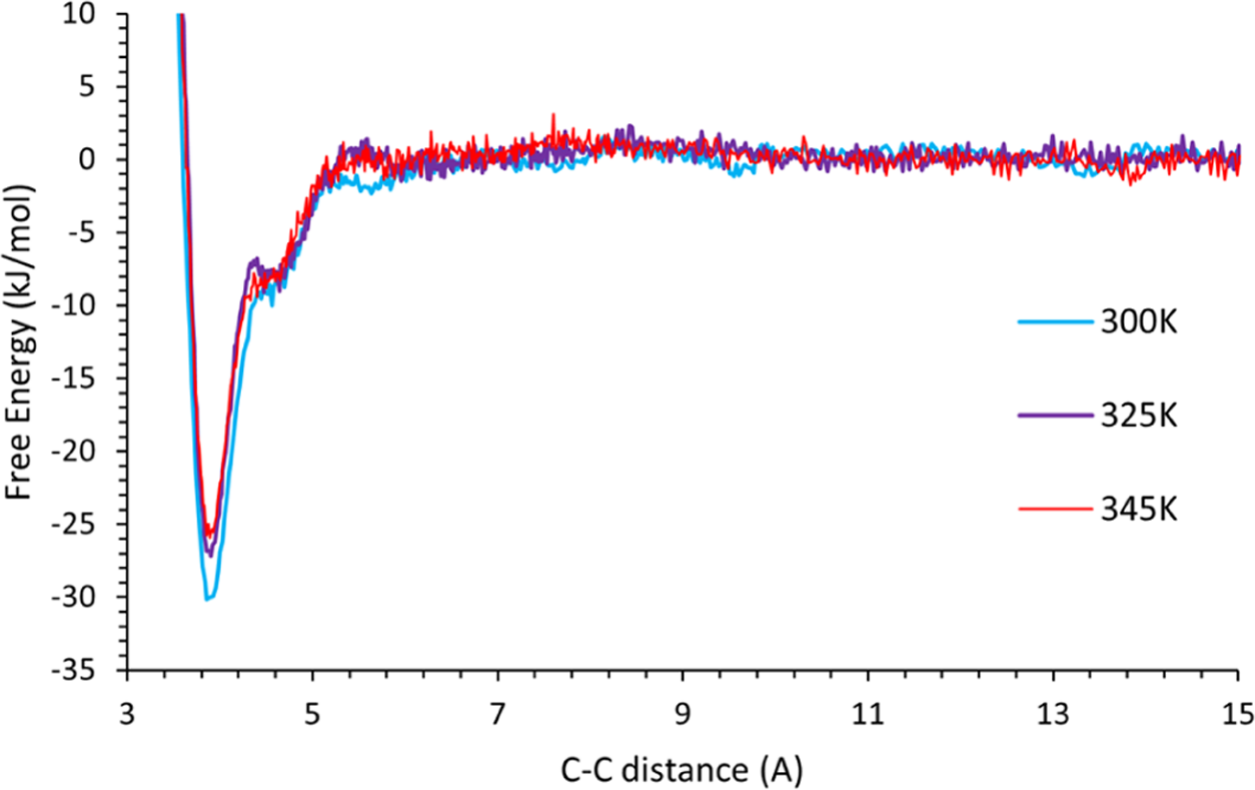
Free energy profiles of two stearic acid molecules self-associating as they approach each other at different temperatures. The depth of the potential well, corresponding to the free energy of dimerization, changes with temperature as expected because the extent of dissociation increases as a function of temperature (eq 4).

The simulations were repeated at temperatures of 300 and 345 K to study the role of temperature on solution association (Figure 4). As expected, the free energy follows the same overall shape; however, the depth of the potential well decreases as a function of temperature.

**Figure 5:**
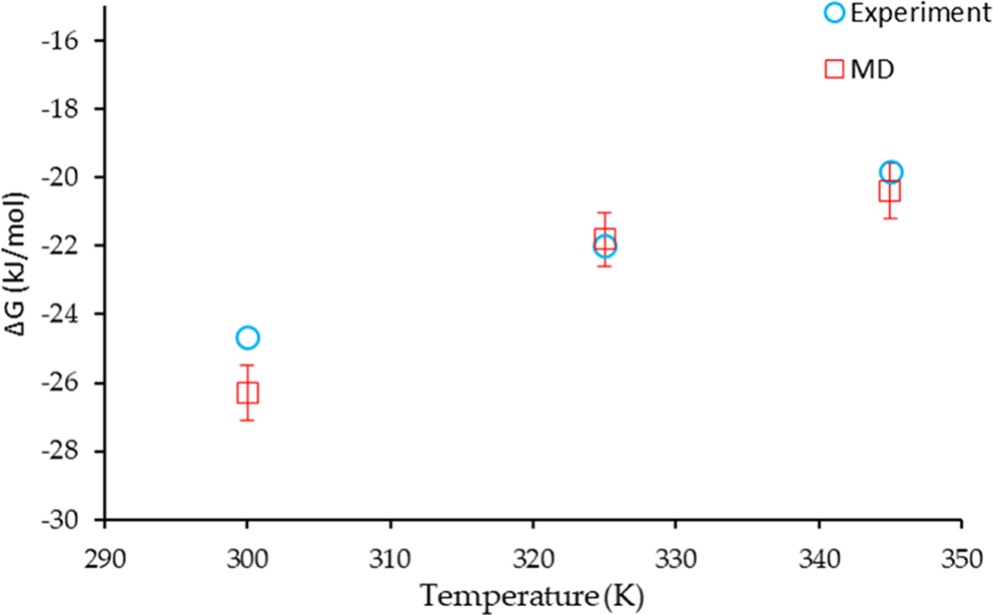
Gibbs free energy of dissociation of two stearic molecules calculated from molecular dynamics and the reported measured experimentally. The simulated data are in reasonable agreement with the experimental results published in our previous paper.^4^ The Gibbs free energy of dissociation increases with temperature due to increased dissociation (i.e., monomer concentration increases, thus *K*_d_ increases, increasing Δ_d_*G*).

As described above, the Gibbs free energy of dimerization at each temperature was numerically calculated by using eq 5. The resultant data are presented in Figure 5 along with the corresponding experimental data reported in our previous paper.^4^ Importantly, there is a very reasonable agreement between the two data sets suggesting that the MD and the experimental approaches support each other, and we can have reasonable confidence in the values of the dissociation constant determined.

### 4.2 Stearic Acid–Water Association

**Figure 6:**
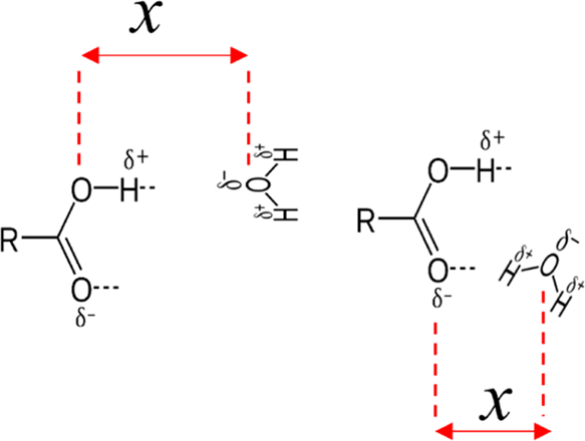
Schematic diagrams of possible association forms of stearic acid and water. (Left) Stearic acid forms a hydrogen bond through its hydrogen atom with the oxygen atom on the water molecule. (Right) The carbonyl oxygen atom forms a hydrogen bond with the hydrogen atoms on the water molecule. In both cases, the separation distance, *x*, is defined as the distance between the relevant oxygen atom on the acid headgroup and the oxygen on the water molecule. δ− and δ+ refer to the partial charges on each atom. It is important to note that while the separation distance is defined through a harmonic force on the center of mass, the rest of the molecules are free; hence, the geometry and orientation of the molecules are not constrained. Therefore, the molecule can adopt the energetically preferred state.

Molecular dynamics simulations were used to calculate the free energy of the stearic acid–water association. The procedure used here was exactly as described for the acid–acid self-association. There are two possible association forms: hydrogen on the water molecule associating with the carbonyl oxygen (CO–water) and the oxygen on the water molecule associating with the hydrogen on the acid headgroup (COH–water). Schematic diagrams of both association forms are presented in Figure 6.

**Figure 7:**
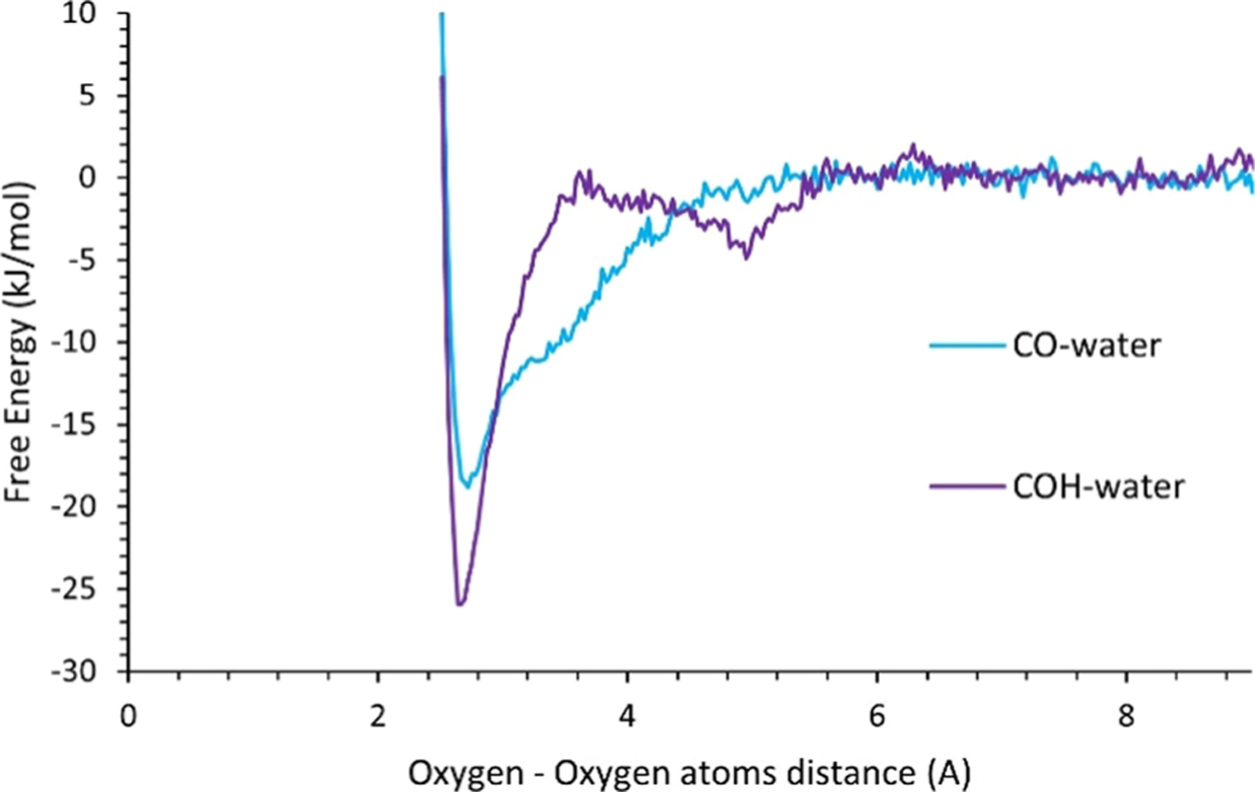
Free energy profile of a stearic acid molecule and water associating as they approach each other at 345 K. The free energy of each association forms (CO–water and COH–water) is presented as a function of O–O separation distance (See Figure 6).

**Figure 8:**
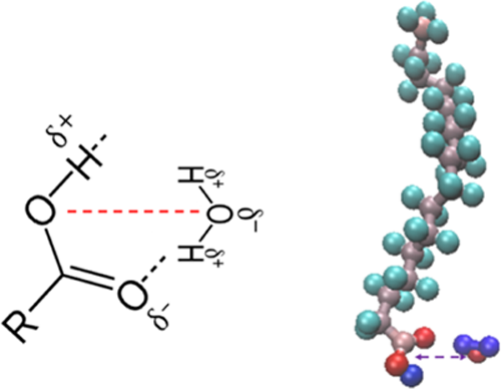
(Left) Schematic of the COH–water association at large separate distances where the H atom on the water molecule has some interaction with the carbonyl oxygen resulting in the second minima present in the free energy profile. (Right) Snapshot from MD simulation at a separation distance of 4.95 A, where the red atoms are oxygen, the blue atoms are water hydrogens, the gray atoms are carbon, and the aqua atoms are CH_2_ and CH_3_ hydrogens. The orientation of the stearic acid and the water molecules suggests some degree of interaction between the carbonyl oxygen and the hydrogen on the water molecule, therefore confirming the interaction suggested in the schematic on the left.

The free energy profiles of a water molecule and a stearic acid molecule associating in both forms (CO–water and COH–water) at 345 K are shown in Figure 7. The free energy profile as a function of the separation distance (defined as the O–O gap, see Figure 6 above) between the associating species remains constant above a separation distance of 6 Å, indicating minimal interaction between the acid headgroup and the water molecule. This defines the “bulk” value of the free energy, set to zero. As the two molecules approach each other further, the profile becomes strongly negative with minima before sharply rising due to the repulsion in the Lennard-Jones potential. The minimum at a distance of 2.7 Å corresponds to hydrogen bonding between the water molecule and the acid headgroup. In the case of COH–water association, there is a second minimum at a distance of 5.0 Å. A possible explanation for this second minima may be the interaction of the carbonyl oxygen and H atoms in water, as schematically shown in Figure 8 (left). In the case of CO–water association, there is insignificant interaction between the O atom on the water molecule and H on the acid headgroup due to the two H atoms on the water molecule “covering” the O atom (i.e., steric hindrance). A cluster analysis was performed on the distance between the carbonyl oxygen and H atoms in water as a function of separation to confirm this (Supporting Information). It is difficult to calculate the extent of the interaction and the corresponding free energy change from the cluster analysis. However, integrating the free energy profile over the second minima only suggests that this additional interaction results in a 0–8% increase in the free energy of dissociation.

The free energy of association was then calculated from the numerical integration of the free energy well using eq 5. The simulations were repeated at temperatures of 300 and 325 K to study the temperature dependence of association. The free energy as a function of temperature is presented in Table 1. The free energy can be used to calculate *K*_d_ using the following relation:
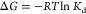
6

**Table 1:** Summary of the Free Energy of Dimerization of Stearic Acid–Water Association as a Function of Temperature and the Corresponding Values of *K*_d0_ and Δ_d_*H*

	COH–water association				CO–water association			
temperature (K)	Δ_d_ G	*K*_d_ (mM)	*K*_d0_ (mM) at 298 K	Δ_d_*H* [kJ/mol]	Δ_d_ G	*K*_d_ (mM)	*K* (mM) at 298 K	Δ_d_ H [kJ/mol]
300	20.71	0.249			18.81	0.912		
323	19.50	0.705	0.225	37.2	17.17	1.677	0.855	24.0
345	18.23	1.741			16.49	3.194		

The *K*_d_ values can be used to calculate *K*_d0_ and Δ_d_*H* by fitting *K*_d_ to eq 4.

## 5 Discussion and Conclusions

In this study, the self-association of stearic acid in cyclohexane as a function of temperature was studied through molecular dynamics simulation. Similarly, the heteroassociation of stearic acid with water was investigated at different temperatures to obtain thermodynamic parameters such as the enthalpy of association. Stearic acid in cyclohexane was found to have a dissociation constant of *K*_d0_ = 0.022 mM at 25 °C. A summary of the simulation results along with experimental results reported in the literature is presented in Table 2. The dissociation constants determined by this study are similar to those determined by others in related solvents.^4,28,30^ Moreover, the data for dimerization in saturated alkanes are especially uncertain, due to the particularly low values of *K*_d0_—a relatively wide range of values from *K*_d0_ = 0.02 to 1 mM have been reported for similar acids in similar alkanes. Therefore, the values obtained for stearic acid from molecular dynamics are within the acceptable range and in good agreement with the experimental values reported in the literature.

**Table 2:** Summary of the Dissociation Constants and the Enthalpy of Dissociation, Including Both the Work Carried Out in This Project and Relevant Data Presented in the Literature^a^

acid	solvent	*T* (°C)	experimental		MD simulation	
			*K*_d_ (mM)	Δ_d_*H* (kJ/mol)	*K*_d*0*_ (mM)	Δ_d_*H* [kJ/mol]
Acid Self-Association in Dry Solvent						
stearic acid	cyclohexane^4^	25	0.0439	57.1	0.0222*	65.7*
stearic acid	hexane^30^	28	0.3463			
stearic acid	hexane^30^	36	4.329	41.003		
stearic acid	hexane^30^	42	5.882			
stearic acid	hexane^30^	48				
stearic acid	toluene^4^	25	0.74	40.5		
stearic acid	heptane^28^	25			0.0240	
stearic acid	hexadecane^28^	25			0.0970	

Acid–Water Heteroassociation						
association form	solvent	*T* (°C)	*K*_h_ (mM)	Δ_d_*H* [kJ/mol]	*K*_h_ (mM)	Δ_d_*H* [kJ/mol]
stearic acid CO–water	cyclohexane^4^	25	0.859	28.6	0.855*	24.0*
stearic acid COH–water	cyclohexane	25			0.225*	37.2*
stearic acid CO–water	toluene^4^	25	19.18	21.2		

a The table is split into ’sections’; the self-association work carried out in this report and other MD studies reported in the literature is on the right-hand side and the experimental data and the corresponding system is presented on the left. All experimental data presented here are from the literature. All data obtained from this work is highlighted through a star (*).

Unfortunately, data for the Δ_d_*H* of acids in hydrocarbons are scarce. For stearic acid in cyclohexane, the experimentally measured value is lower than that simulated with MD. In our recent published work,^4^ a theoretical prediction based on the classical Onsager–Böttcher polar cavity model with the Flory–Huggins correction factor predicts a higher value than experimentally measured. One possible reason for this is that many of the experimental results published in the literature assume a system with closed dimers only (i.e., 2 hydrogen bonds per dimers), and actually the value of Δ_d_*H* is for some mixture of open and closed dimers, rather than just closed dimers. Therefore, this might explain the discrepancy between the two values, but more importantly, it enables the calculation of the percentage of open dimers that is not possible to determine from the IR data alone because it is not possible to accurately deconvolute the separate bands corresponding to open and closed dimers. To test this hypothesis, using the experimental data and models reported in our recent paper,^4^ the value of Δ_d_*H* can be fixed to the MD value and apply the open dimer model to the data for dry cyclohexane (the details of this calculation are provided in the SI and a recent paper). The results confirm our hypothesis: fitting the experimental data with the same 4-parameter model but constraining the enthalpy of dissociation to the value obtained from MD results in a similar goodness of fit despite one less degree of freedom. With this more constrained fit, we are able to give a percentage of open dimers for stearic acid in cyclohexane which are estimated to be∼ 1.5% at 25 °C and ∼4% at 70 °C. Importantly, these are similar to the values obtained by a combination of IR and the classical Onsager–Böttcher polar cavity model.^4^ This suggests that MD, by providing a more constrained fit, enables a more detailed study of solution association.

The association of acid–water is rarely studied, with the only reported study found in the literature being our recent paper,^4^ making it extremely difficult to compare the data presented with others. The stearic acid CO–water association in cyclohexane measured experimentally is in reasonable agreement with the molecular dynamics simulation. The values are a little lower than that of stearic acid CO–water association in toluene reported in the literature; however, the chemical nature of the solvent present in the system is also important when considering the degree of association. Solvents such as toluene (dielectric constant: 2.38) have higher dielectric permittivity than alkanes such as cyclohexanes (dielectric constant: 2.02), preferentially stabilize the unassociated form of the acid forcing the equilibrium toward dissociation (higher *K*_d0_), more than straight-chain hydrocarbon solvents such as heptane.^24^ Therefore, when the effect of the solvent is considered, the values are within the acceptable range. MD simulations were also used to study the association of water with the OH part of the stearic acid from the free energy profile. The simulation method indicates significant interaction between the H atom on the water molecule and carbonyl O atom on the acid headgroup at large separation distances. Therefore, the MD method used in this report is likely to have overestimated the OH-water association constant and the corresponding enthalpy of association, as presented in Table 2. Analysis of the free energy profile suggests that this additional interaction results in a 0–8% increase in the free energy of dissociation.

## Supporting Information Available

Brief discussion of the details of combination of MD and FTIR results that enable the proportion of open dimers to be calculated; sensitivity analysis and cluster analysis of water–acid association that provide justification for the time scale of the simulation and insight into the shape of the free energy profile presented in the main text (PDF)

## Notes

For the purpose of open access, the author has applied a Creative Commons Attribution (CC BY) license to any Author Accepted Manuscript version arising from this submission.
