# Supplementary material for: Molecular Dynamics: Investigating the Self-Association of Stearic Acid and Heteroassociation of Stearic Acid–Water in Cyclohexane
Source: ACS Omega. 2023 Oct 25;8(44):41100–6. doi: 10.1021/acsomega.3c03473 (PMC10633822; doi:10.1021/acsomega.3c03473)
Supplement: Supplementary file 1 — ao3c03473_si_001.pdf [file ao3c03473_si_001.pdf]

Molecular Dynamics: Investigating the Self-Association of Stearic Acid and Hetero Association of Stearic Acid-Water in Cyclohexane

Najib Sharifi

Institute for Energy and Environmental Flows and Department of Chemistry, University of Cambridge, Cambridge, UK

Address for correspondence: ns650@cam.ac.uk

Supplementary information

Using the model presented in our previous paper[4], using the optimum model (M04 in Table S1) the fitting is repeated but the enthalpy of dissociation is fixed to that obtained from MD simulations. The result fitted parameters are shown in grey (last row, M13 in Table S1), where the dispersion is essentially the same as that obtained from M04.

In our previous paper, a regression was performed to fit experimental data on solution association of stearic acid at a range of temperature and concentrations simultaneously. A range of different models (with different permutation and number of fitted parameters) were used to fit the experimental data. Table S1 below outlines the models and the fitted values with the 'goodness of fit', the minimal value of the standard deviation σ. The criterion for the best model is defined as one that has (i) minimal standard deviation among all variants with the same number p of independent parameters and (ii) no substantial drop (compared to the experimental uncertainty) in the standard deviation upon addition of another free parameter.

The concluded best model is the 4-parameter model (M04 in Table S1) which does not account for open dimers. The results suggest that the data is not sensitive to the fraction of open dimers, x_o. Further analysis of the IR lineshape of the peaks in the FTIR data, also suggests that additional peaks that might indicate the presence of any open dimer cannot be identified. Nevertheless, there is a significant fraction of open dimers, especially at higher temperature, based on analysis of the values of the heats of dissociation in Table S1. The reason is that the effects from the two parameters x_o and ε_T are difficult to distinguish, and lower x_o can be compensated by a decrease in $\Delta_d H$ and increase in ε_T, i.e. ε_T and x_o cannot be determined independently from the FTIR data without extra information.

Table S1. In our previous paper[4] a systematic study was carried out to identify a suitable model to describe the self-association of stearic acid in cyclohexane. These models were all used to fit the experimental data and the model with the lowest number of fitted parameters that resulted in a statistically good fit is model M04 where 4 parameters are fitted. Using the same model, with the enthalpy fixed to that obtained from molecular dynamics results in a similar dispersion as M04 model. K_d^o is the dimerization constant at 25°C, $\Delta_d H$ is the enthalpy of dissociation, ε_m and ε_d are the monomer and dimer IR extinction coefficients from the Beer-Lambert law. ε_{mT} and ε_{dT} are the corresponding temperature coefficient for ε_m and ε_d. x_o is the fraction of open dimers and $\Delta_o H$ is the enthalpy of open dimers.

Number	Model	K_d°(mM)	Δ_dH [kJ/mol]	ε_m	ε_d	ε_{mT}	ε_{dT}	n	x_o°	Δ_oH [kJ/mol]	σ	p Indep. Para
M01	Closed Dimer	0.0270	72.3	0.073	ε_m	0	0	1	N/A	N/A	0.0182	3
M02	Open Dimer	0.093	79.8	0.042	ε_m	0	0	1	3.1 %	Δ_dH/2	0.0178	4
N03	Closed Dimer	0.131	57.8	0.042	0.079	0	0	1	N/A	N/A	0.0102	4
M04	Closed Dimer	0.0439	57.1	0.077	ε_m	1.12	ε_{mT}	1	N/A	N/A	0.0063	4
M05	Open Dimer	0.0439	59.1	0.077	ε_m	1.10	ε_{mT}	N/A	1.6%	Δ_dH/2	0.0062	5
M06	Closed Dimer	0.0396	57.3	0.081	0.077	1.17	ε_{mT}	1	N/A	N/A	0.0064	5
M07	Open Dimer	0.0366	56.3	0.077	ε_m	1.11	ε_{mT}	N/A	0.87%	15.00	0.0063	6
M08	Closed Dimer	0.0492	56.8	0.081	0.079	1.743	1.103	1	N/A	N/A	0.0021	6
M09	Closed Dimer	0.0494	65.5	0.090	0.079	0.485	0.100	13.9	N/A	N/A	0.0021	7
M10	Open Dimer	0.0546	53.2	0.072	0.080	1.123	ε_{mT}	N/A	0.007	Δ_dH/2	0.0017	7
M11	Open Dimer	0.0466	56.7	0.079	0.079	1.667	1.074	N/A	0.006	Δ_dH/2	0.0021	7
M12	Open Dimer	0.0359	61.7	0.081	0.079	1.884	1.044	N/A	0.016	15.00	0.0018	8
M13	Open Dimer	0.0222	65.7	0.076	ε_m	1.04	ε_{mT}	N/A	1.53%	Δ_dH/2	0.0064	3

Sensitivity analysis

After each step of 0.5 Å, further equilibration is required, followed by the umbrella sampling analysis. A sensitivity analysis was carried out to find the time required for a typical umbrella run. Four sets of simulations were performed with different amounts of time; 0.2 ns, 1 ns, 2 ns, and 4 ns with 1/4 of the total time used for equilibration and 3/4 of the time used for umbrella sampling analysis at each step. The exact shape and details of the free energy profile is discussed in the main paper, here, we only consider changes in the profile as a function of the total run time (Fig. S1). Increasing the time for the umbrella from 0.5 ns to 2 ns results in the free energy profile changing, however, increasing the umbrella run time to 4 ns results in a small change in the energy profile. The corresponding ΔG_{dim} calculated (equation 5) from each run is given in Table S2 which shows no changes between the 2 ns and 4 ns runs, suggesting using a total time of 2 ns for each umbrella run is sufficient. Any further increase in simulation time results in no significant changes in the free energy profile but results in a significant rise in computational cost and time. This is also reflected in the histogram analysis, where for 0.5 ns, the histograms are not converged and but show good statistical convergence at 2 ns (supplementary information).

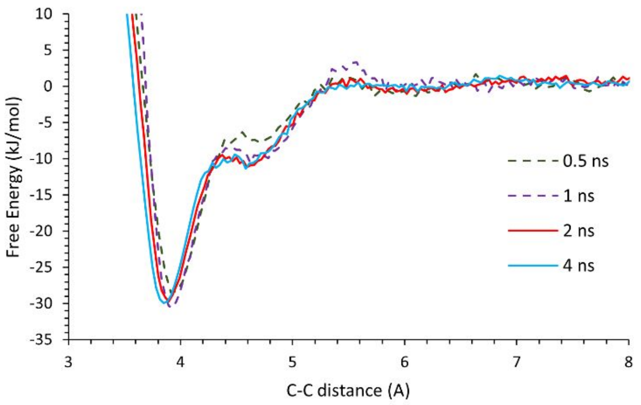

Figure S1. Free energy profile of 2 stearic acid molecules self-associating, as a function of the umbrella time. Using a total time of 4 ns for each umbrella run shows no significant changes compared to 2 ns, therefore, 2 ns is taken to be sufficient.

Table S2. The change in ΔG_{dim} as a function of the total simulation time for each step. The free energy fluctuates strongly with simulation time below 2 ns, however, remains constant above 2 ns.

Simulation time (ns)	ΔG_{dim} (kJ/mol)
0.5	-24.5
1	-29.8
2	-26.4
4	-26.4

In addition to the final free energy profile as a function of reaction coordinate as presented in the main text of the paper, in determining the simulation time required for statistical convergence, the histograms can be used to check for convergence. The histograms as a function of the reaction coordinates for a simulation time of 0.5 ns and 2 ns are presented in Fig. S2. For the 0.5 ns, at large C-C distances, the histograms capture the general Gaussian histograms shapes, however, there is significant noise in the profile suggesting the profiles have not converged. Whereas in the case of each umbrella window simulated for 2 ns, the simulations yield well-converged statistics as visible from the Gaussian histograms at a large C-C distances.

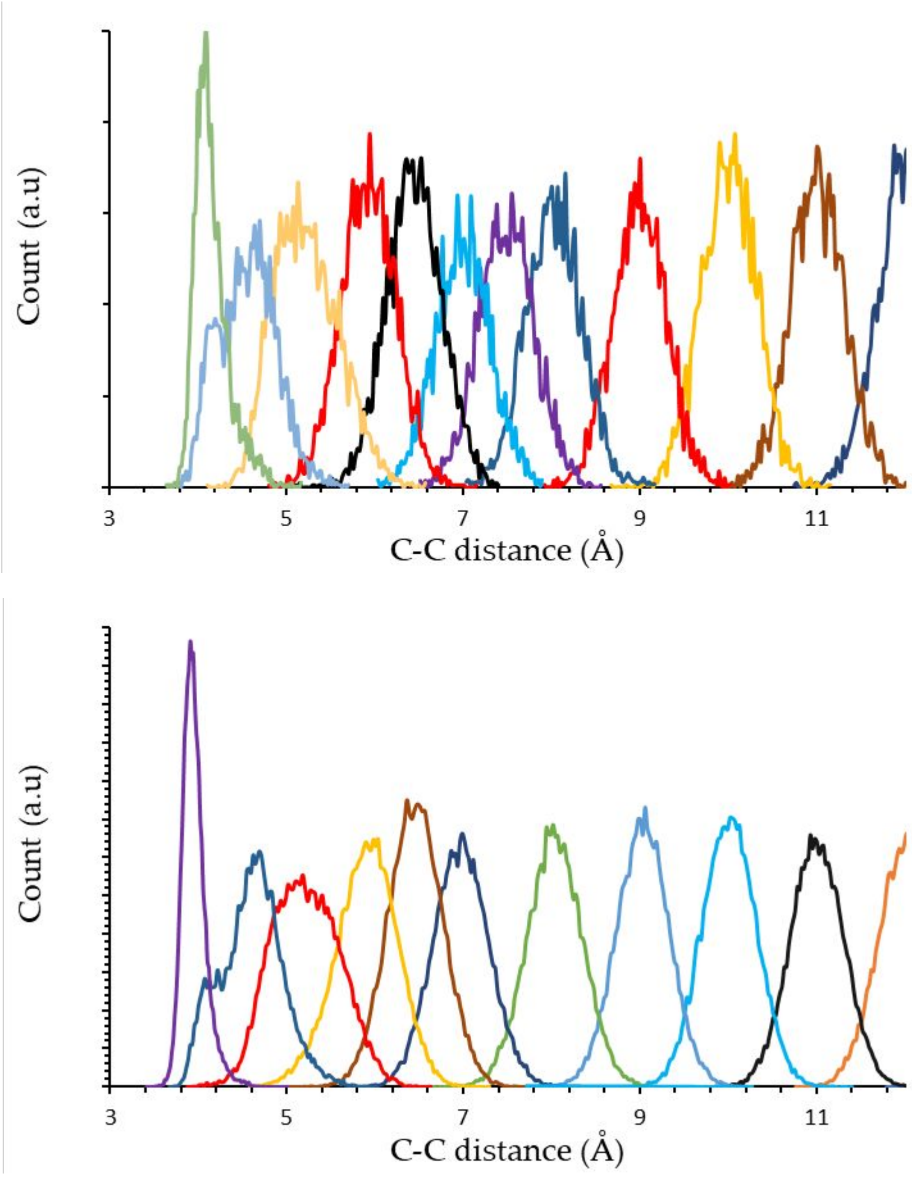

Figure S2. The resultant umbrella histograms from 0.5 ns (top) and 2 ns (bottom) simulation time per umbrella window, showing good overlap between neighbouring histograms.

Cluster analysis of acid-water association

A cluster analysis of the RMSD, at a separation of around 4 -5 Å, between carbonyl oxygen and H atoms in water was carried to estimate the occurrence of hydrogen bonding. This is done by analysis of the RMSD and the distance along the reaction coordinate every 50 ps (Fig. S3). Using a limit of 3.3 Å for hydrogen bond length, the percentage RMSD within this limit is approximately 60% around separation distance of 4 Å. More care and sophisticated statistical analysis is required to get a more accurate estimation of this interaction occurrence, however, the aim here is to determine whether there are hydrogen bonding occuring between carbonyl oxygen and H atoms in water, therefore, the analysis here is only confirmation of this hypothesis.

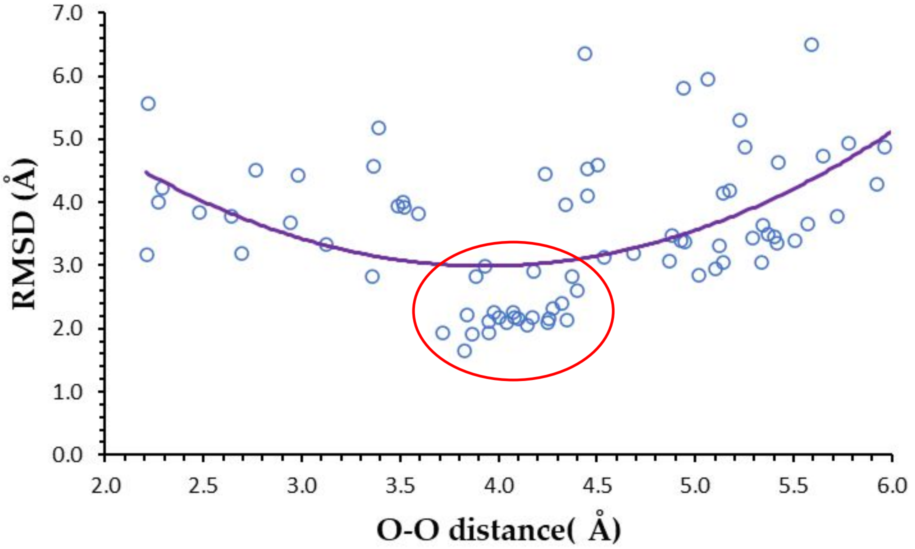

Figure S3. RMSD between carbonyl oxygen and H atoms in water as a function of reaction coordinate, a second degree polynomial fit is also presented as a guide to the overall shape. Around 4 Å, a cluster with low RMSD is present suggesting some interaction between carbonyl oxygen and H atoms in water as discussed in the main text of the paper.
